# Educational inequalities in employment of Finns aged 60–68 in 2006–2018

**DOI:** 10.1371/journal.pone.0276003

**Published:** 2022-10-17

**Authors:** Anu Polvinen, Aart-Jan Riekhoff, Satu Nivalainen, Susan Kuivalainen

**Affiliations:** Finnish Centre for Pensions, Helsinki, Finland; Stanford University, UNITED STATES

## Abstract

The aim of this study was to explore the employment of 60–68-year-old men and women by educational level over the period 2006–2018 and the magnitude of educational inequalities in employment. We used individual-level register data from Statistics Finland including all Finns aged 60–68 over a period of 13 years. In addition to calculating employment rates for men and women by educational levels, we estimated the relative index of inequality (RII) and slope index of inequality (SII) to measure the magnitude of relative and absolute educational inequalities in employment. The results show that the employment rates increased in all educational levels over the period 2006–2018. Relative educational inequalities in employment remained stable mainly among the 63–65-year-olds but decreased among the 60–62-year-olds and the 66–68-year-olds. However, absolute educational inequalities in employment increased in all age groups for both men and women.

## Introduction

Extending working lives has been an important aim of public policy in recent years. In many European countries, labour force participation of those aged 60 years and older has increased while statutory retirement ages are being raised and early retirement has become less attractive and more restricted [1–3]. Nevertheless, policymakers are still looking for improvements as employment rates of those older than 55 often continue to be below the average employment rates. There is indeed a substantial group in the population older than 60 that wants to continue working while enjoying good health and work ability [4–8].

However, ‘older workers’ are not a homogenous group and there is no one-size-fits-all policy that would further raise the employment levels of all [9]. First, the factors that push and pull people out from work tend to be reinforced when people age. Most studies and statistics show the employment rates of older workers in rather broad age brackets, for example, 55–64 or 60–64 years. Using these broad age brackets obfuscates the effects of (biological) ageing and events during this stage of the life course. For example, the risk of poor health increases with age while the retirement of a spouse, having grandchildren or having a family-member who falls ill might suddenly change the preference to continue working. Moreover, the incentives for work and exit are linked to eligibility rules in social security and, in particular, in pension systems at different ages.

Second, especially low-educated workers and people from more disadvantaged socioeconomic groups are at risk of missing the tide of extended working lives [10–12]. Socioeconomic and educational inequalities in early exit from working life are well known in previous literature [13–18]. Low-educated workers tend to leave the labour market earlier than the high-educated, which creates educational inequalities in the employment rates of older workers. Yet relatively little is known about how large these inequalities are and how they have developed across time. If the employment rates of older workers are rising but educational inequalities remain stable or increase, policymakers may need to reconsider ways of making the extension of working lives more inclusive.

Studying educational inequalities in the employment of older workers across time is complicated by the fact that the educational structure of the population continues to change. Cohorts that now approach the retirement age have a considerably higher education than cohorts that retired fifteen years ago. This educational expansion has had a positive effect on extending working lives [1]. However, while it is possible that the overall inequalities between older workers in society have declined because the share of high-educated people has increased, the relative disadvantages for the low-educated groups may have remained the same or even grown in younger cohorts.

Finland makes an interesting case for studying educational inequalities in employment for several reasons. While the employment rate in the age group 55–64 years was only around 35 percent in the mid-1990s, it has increased to more than 60 percent in recent years. A series of pension reforms during this period explicitly aimed at extending working lives and increasing the employment of older workers. At the same time, over the past decades, the share of people who have completed tertiary education has risen drastically in this age group while the share of those with low qualifications continues to decline (see S1 Table in S1 File). Currently, Finland is one of the countries in Europe with the most highly educated older workforce. However, at the present, there is no information on whether the rise in the employment rate has occurred in all pre- or post-retirement-age groups and on all educational levels. Finland could forebode of how the educational inequalities in employment of older people will develop in other countries.

This study explores employment of 60–68-year-old men and women by educational level over the period 2006–2018 and the magnitude of educational inequalities in employment. We propose a novel method to analyse both absolute and relative educational inequalities in employment. Using exceptional register data for the entire Finnish population allows us to observe employment rates in smaller-than-usual age brackets and to disaggregate these by gender and level of education. We analyse employment levels and educational inequalities among three age groups: 1) those that are approaching their retirement age (60–62 years), 2) those that are eligible for an old-age pension but have incentives and often good opportunities to continue working (63–65 years), and 3) those that possibly face increasing obstacles and disincentives to continue working (66–68 years). We observe trends in employment and educational inequalities in these age groups between 2006 and 2018.

This article is organised as follows. The following section introduces previous literature on educational inequalities in employment at older age and describes changes in the incentives for employment at older age in the Finnish pension system. Section 3 describes the data and methods. Section 4 presents our findings, which are discussed further in section 5.

## Background

### Educational inequalities in employment at older age

Studies have identified numerous mechanisms behind the early exit and lower employment rates of low-educated older workers. First, a lower socioeconomic status is associated with poorer health, which may limit possibilities to continue work [17–25]. Moreover, De Breij et al. [13] found that poor health increases the risk of early exit to a greater extent among the low-educated than the high-educated. As a result, those with a low socioeconomic status are more vulnerable to receive sickness benefits [26–28] and disability retirement [29–32].

Second, lower education is associated with work in occupations and sectors that are more prone to early exit, while those with higher education are more likely to work in sectors and occupations where extended working lives are possible and encouraged. The low-educated tend to be found in jobs with weaker and straining work conditions and less autonomy, both of which have been found to increase the risk of early exit, also because of the effects these conditions have on one’s health [33, 34].

The low-educated are also more likely to be employed in sectors that are vulnerable to economic shocks and restructuration, increasing the risks of (long-term) unemployment and early exit [15, 35]. Studies on the impact of the Great Recession on the employment of older workers showed that the low-educated were more likely to lose their jobs and less likely to be reemployed [36–38].

Additionally, low-educated workers are less likely to invest in training than their high-educated peers [39] yet their wages may increase in seniority-based wage systems [40], making them less attractive and often relatively more expensive for employers to retain or employ [41]. The high-educated, on the other hand, might possess skills and knowledge that are valuable to employers and are therefore more actively retained.

Third, the pull factor of the pension system and early exit pathways is likely to be greater for the low-educated. Higher education tends to be associated with higher earnings, making the marginal gains of staying employed in the late career greater for the high-educated. For the less educated with lower earnings (but with sufficient pension entitlements), an earlier exit might be more attractive since their pensions and other social security benefits are often more generous in relation to what they would earn by continuing to work [42]. This is the case especially in pension systems like the Finnish that include a minimum or guarantee pension which reduces the risk of falling into poverty after retirement. The lower marginal gains of continued working among the low-educated might also mean that they are more likely to retire in case a spouse falls ill or if they become grandparents [43, 44].

Previous studies have shown socioeconomic inequalities not only in early exit but also in old-age retirement. While people with a lower socioeconomic status often exit working life through unemployment or disability before reaching their old-age retirement age, upper-level employees more likely transfer to old-age retirement directly from work [45]. Moreover, those with a lower socioeconomic status tend to retire on an old-age pension as soon as it is possible while the majority of upper-level employees and high-educated workers stay on at work longer [46, 47].

However, old-age retirement does not necessarily mean that working will end. Working alongside an old-age pension has become more popular in many countries in recent years [4, 48–51]. Previous studies have found that people who continue working while receiving an old-age pension more often have a higher socioeconomic status, a higher educational level and better health than those who do not continue working [8, 49, 52, 53].

### Pension system reforms and late-career employment in Finland since 2005

At least part of the rise in employment of older workers in Finland can be attributed to the way the pension system was reformed in 2005 and 2017. Their main means to raise employment levels at the tail-end of working life are summarised in Table 1. Since 2005, the retirement age of Finnish salaried employees and the self-employed is flexible, between 63 and 68 years. The age of 63 has been the most popular age for old-age retirement after the 2005 reform although working beyond the minimum retirement age was rewarded with a higher pension accrual rate. In 2017, the lower age limit for old-age retirement started to rise by three months for each age cohort until it is 65. At the same time, the possibilities for early exit (i.e., before the minimum statutory retirement age) became substantially limited (see also Table 1). Currently, the only real early exit options are the disability pension for those with considerable work-related health problems and the partial old-age pension which can be drawn from the age of 61.

**Table 1:** Overview of relevant pension reforms in Finland 2005–2017.

Reform year	Reform	Cohorts affected
2005	Old-age pensions: Introduction of flexible retirement age between 63 and 68 instead of fixed at 65.	1942 ->
	Individual early retirement pension: Abolished (previously available for disability reasons at 60 [private sector] and 59 [public sector]).	1943 ->
	Early old-age pensions: Lower age limit raised from 60 (private sector) and 58 (public sector) to 62.	1945 -> (private sector) and 1947 -> (public sector)
	Unemployment pensions: Abolished (previously available at age 60 after extended period of unemployment benefits). For the long-term unemployed born before 1958, old-age retirement possible at age 62.	1950 ->
2013	Early old-age pensions: Possibility to retire early at age 62 with a permanent reduction in pension benefits abolished.	1952 ->
2017	Old-age pensions: Lower age limit starts to rise by three months per age cohort until it is 65.	1955 ->
	Introduction of partial old-age pension: Possibility to take part of the pension at age 61.	1949 ->

Source: etk.fi, Riekhoff (2018)

Many studies have shown that there are educational or socioeconomic differences in labour market exit and retirement in Finland. As a rule, the low-educated are found to be at a higher risk of early exit from the labour market [41, 53]. The high-educated workers (especially women) tend to work longer and take out their old-age pension later [54, 55]. Additionally, studies have documented a considerable social gradient in the risk of retirement on a disability pension [29, 56]. The first studies on the more recently introduced partial old-age pension suggest that the take-up of this pension is more often associated with unemployment which is more common among the low-educated [57, 58].

Nowadays, approximately 14 percent of Finnish old-age pensioners aged 63–74 continue working while they receive an old-age pension [51]. Also, a previous Finnish study has found that circa 14 percent of full disability pensioners and almost 80 percent of partial disability pensioners engage in paid work [59]. The same study also found that high-educated people work while on a disability pension more often than the low-educated. A majority of those who receive a partial old-age pension continues to work: only one third of the employed claimants have reduced their working hours after claiming a partial old-age pension [57, 58].

## Data and methods

This study is based on individual-level register data of the total population of Finland from 2006 to 2018. The data were combined from several registers collected by Statistics Finland and included all Finnish men and women aged 60–68 over a 13-year period from 2006 to 2018. The cohorts included in this study were born between the years 1938 and 1955. The annual number of observations was 516,546 in 2006; 600,955 in 2009; 656,897 in 2012; 665,033 in 2015 and 636,735 in 2018.

### Age

We studied employment and educational inequalities in three different age-groups. The groups were divided into people who were (a) approaching their retirement age (60–62 years), (b) eligible for an old-age pension but had incentives and often good opportunities to continue working (63–65 years), and (c) facing increasing obstacles and disincentives to continue working (66–68 years).

### Employment

Employment was measured by income derived from paid work or self-employment in each year of the study. This method can identify employment even if it is short-term or the earnings are low. The register data include all income received from paid work or self-employment with no missing data. Income is based on tax-registers. Typically, employment is measured within a relatively short period, for example, in the previous week or last calendar day of the year, leaving out all other employment. Moreover, employment status variables only make a crude distinction between being employed or being a pensioner, which excludes the possibility of working in retirement. We assessed an individual as employed if their annual earnings were more than 5,000 euros in 2006. The limit of 5,000 euros can be considered to represent a relevant level of annual income from work or self-employment to define employment. On the one hand, it is sufficiently low to detect relatively short spells of work against low wages. On the other hand, it is high enough to exclude cases where work was rather marginal. The limit also roughly equals an average two-month salary. The earnings limits for employment were adjusted with the wage-coefficient for each year: 5,607 euros in 2009; 6,072 euros in 2012; 6,411 euros in 2015; and 6,543 euros in 2018.

We also performed sensitivity analysis with lower and higher euro-limits for employment (see the Appendix). In these analyses, the lower earnings limits were 2,000 euros in 2006. In the analysis concerning the upper earnings limits, the limit was 8,000 euros in 2006. The lower earnings limit (2,000 euros in 2006) can indicate quite little annual employment while the higher limit (8,000 euros in 2006) indicates higher annual incomes received from paid work or self-employment.

### Education

Educational level was classified into primary school (up to 9 years of education), secondary education (up to 12 years of education), lower tertiary education (up to 15 years of education) and higher tertiary education (16+ years of education).

### Statistical methods

Employment rates by education were calculated separately for men and women in three different age groups (60–62 years, 63–65 years and 66–68 years) and by three-year intervals over the period 2006–2018. Employment rates were presented as percentages.

We calculated the relative index of inequality (RII) and slope index of inequality (SII) and their 95-percent confidence intervals to measure the magnitude of relative and absolute educational inequalities in employment, respectively. This was done separately for men and women in three age-groups over the study period. RII and SII are methods used to compare inequalities over time or across populations [60, 61]. The methods are based on regression modelling. They take into account the sizes of the subgroups, which obviously are changing due to educational expansion. Educational levels are transformed into summary measure ridits that are scaled from zero to one. The population in each educational levels is disposed a ridit score based on the midpoint of the range in the cumulative distribution of the participants. For example, if the share of primary-educated is 16 percent, it is assigned a ridit score of 0.08 (0+0.16/2). If the share of the next educational level is 30 percent, it is assigned a ridit score of 0.31 (0.16+0.30/2), and so on. A one-unit change in ridit is equivalent to moving from the bottom to the top of the educational distribution. RII and SII are obtained by regressing the employment status on ridit. SII and RII and their 95-percent confidence intervals are calculated using generalised linear models (log-binomial regression). A logarithmic link function is used for RII and an identity link function for SII.

RII compares the employment rates of the extremes of the educational distribution in relative terms while taking all educational levels into account. RII values over 1.00 address educational inequalities in employment. The RII can be expressed as a percentage by subtracting 1 and multiplying by 100. For example, a RII of 1.5 indicates a 50-percent higher risk of employment among the high-educated than among the low-educated [62]. SII values, on the other hand, indicate the magnitude of absolute educational inequalities and measure the prevalence difference. Values over 0.00 show educational inequalities in employment.

It is relatively common that absolute inequalities and relative inequalities move in different directions [61]. For example, when employment increases with the same percentage for the people of all education levels, absolute inequalities will increase while relative inequalities will remain the same [62]. Especially when the occurrence of a state is less common, such as employment in the oldest age group, relative inequalities tend to be high and absolute inequalities low. Therefore, it is important to look at both RII and SII simultaneously when studying educational inequalities in employment at older ages. RII and SII are much used and well-known measures especially in the study of social inequalities in health [60, 61, 63–65]. In this study, we broaden the measurements of relative (RII) and absolute inequalities (SII) to study educational inequalities in employment.

We calculated the interaction terms of ridit and gender (ridit*gender) to describe RII and SII differences for men and women in each year. We also calculated the interaction of ridit and time (ridit*time) and the interaction of ridit, time and gender (ridit*time*gender). The interaction terms and their p-values estimate whether the time trends of RII or SII are statistically significant. The calculations were made at three-year intervals, so that the time points were 2006, 2009, 2012, 2015 and 2018.

## Results

**Fig 1:**
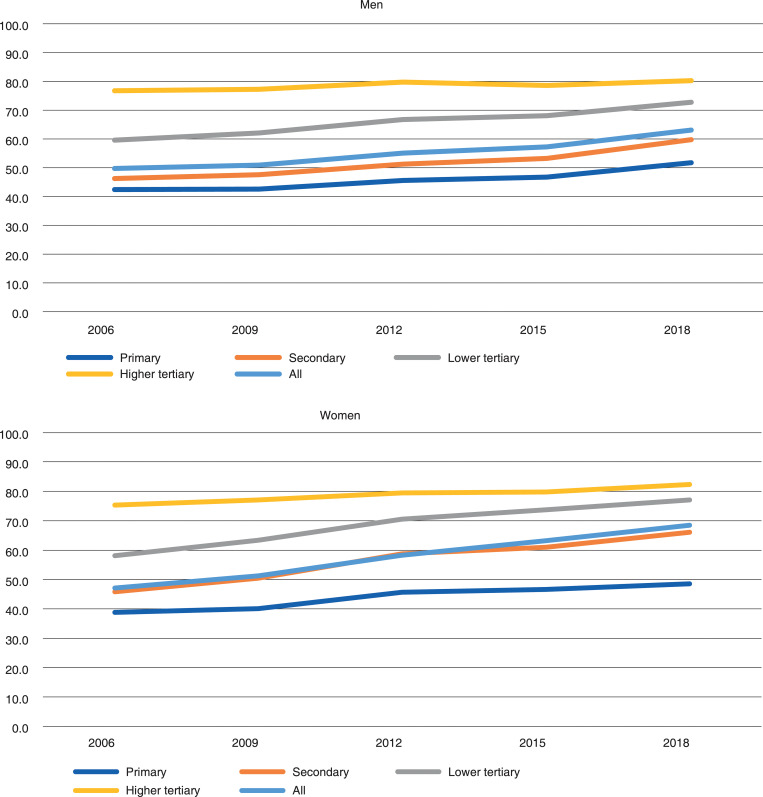
Employment rate of 60–62 year-old men and women in Finland in 2006–2018, by educational level.

Employment increased in all educational levels among men and women aged 60–62 over the period 2006–2018 (Fig 1). In 2006, the employment rate was 42 percent among men and 39 percent among women with a primary education. In 2018, the corresponding figures were 52 and 49 percent. Among women with a higher tertiary education, the employment rate was 75 percent in 2006 and 82 percent in 2018. The corresponding figures for men were 77 and 80 percent.

Educational inequalities in employment were evident among 60–62-year-old men and women over the period 2006–2018, both in relative and in absolute terms (Table 2). Differences in inequalities between men and women were relatively small yet statistically significant in most years, as exemplified by the p- values for the gender*ridit interaction terms. Both for men and women we observed a continuous year-by-year decline in relative educational inequalities in employment: from 2.03 in 2006 to 1.73 in 2018 for men and from 2.12 to 1.69 for women for the same year. The time*ridit for both genders and time*ridit*gender interaction terms were statistically significant. Absolute educational inequalities in employment, however, increased between 2006 and 2012 for men and between 2006 and 2009 for women, after which the values declined or at least stabilised between 2015 and 2018 at a slightly higher level than in 2006.

**Table 2:** Educational inequalities in employment among 60–62-year-old men and women. Relative index of inequality (RII), Slope index of inequality (SII) and their 95-percent confidence intervals (95% CI).

	Men					Women				
	2006	2009	2012	2015	2018	2006	2009	2012	2015	2018
RII	2.03	2.07	2.01	1.92	1.73	2.12	2.16	1.90	1.81	1.69
95% CI	1.98–2.08	2.03–2.11	1.97–2.05	1.88–1.95	1.71–1.76	2.07–2.17	2.11–2.20	1.88–1.94	1.78–1.83	1.67–1.72
Gender*ridit (estimate & p-value)						-0.0454	-0.044	0.0534	0.0587	0.0245
						p = 0.0064	p = 0.0025	p = < .0001	p = < .0001	p = 0.0306
Time*ridit (estimate & p-value)					-0.0139					-0.0229
					p = < .0001					p = < .0001
Time*ridit*gender (estimate & p-value)										-0.0115
										p = < .0001
SII	0.332	0.350	0.368	0.362	0.344	0.340	0.386	0.387	0.387	0.373
95% CI	0.321–0.343	0.340–0.360	0.357–0.378	0.351–0.372	0.333–0.354	0.329–0.351	0.376–0.396	0.377–0.397	0.378–0.397	0.363–0.383
Gender*ridit (estimate & p-value)						-0.0081	-0.0360	-0.0196	-0.0255	-0.0291
						p = 0.3055	p = < .0001	p = 0.0078	p = 0.0005	p = < .0001
Time*ridit (estimate & p-value)					0.0015					0.0012
					p = 0.0094					p = 0.0241
Time*ridit*gender (estimate & p-value)										-0.0096
										p = < .0001

**Fig 2:**
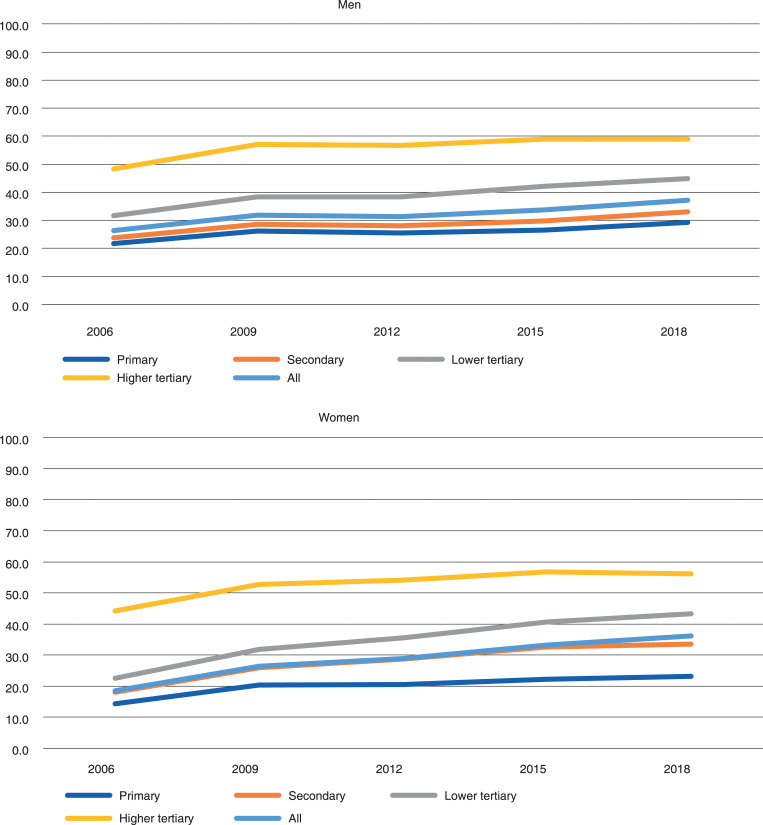
Employment rate of 63–65-year-old men and women in Finland in 2006–2018, by educational level.

Also, among 63–65-year-old men and women employment increased on all educational levels over the study period 2006–2018 (Fig 2). In 2006, the employment rate was 22 percent for men and 14 percent for women with a primary education. In 2018, the equivalent figures were 29 and 23 percent. Among women with a higher tertiary education, the employment rate was 44 percent in 2006 and 56 percent in 2018. The corresponding figures for men were 48 and 59 percent.

Table 3 shows that educational inequalities in employment were also considerable among 63–65-year-old men and women. Again, gender differences were present in almost all years. Especially relative inequalities were larger in this age group than in the younger age group of 60–62-year-olds. Among men aged 63–65, we observed a temporary increase in relative inequalities between 2009 and 2015, but by 2018, the RII returned to a similar level as in 2006. The coefficient for the time trend was not statistically significant for men. Among women there was a slightly decreasing trend in relative inequality, but the confidence intervals of RII for each of the years suggest that inequalities did not vary substantially. Absolute values of inequality, on the other hand, increased steadily. While SII increased between 2006 and 2018 from 0.215 to 0.295 among men, the numbers almost doubled, from 0.179 to 0.342, among women.

**Table 3:** Educational inequalities in employment among 63–65-year-old men and women. Relative index of inequality (RII) and Slope index of inequality (SII) and 95-percent confidence intervals (95% CI).

	Men					Women				
	2006	2009	2012	2015	2018	2006	2009	2012	2015	2018
RII	2.41	2.41	2.49	2.61	2.42	2.81	2.61	2.78	2.69	2.70
95% CI	2.30–2.51	2.33–2.49	2.41–2.57	2.53–2.69	2.34–2.49	2.66–2.96	2.51–2.71	2.69–2.88	2.61–2.78	2.62–2.79
Gender*ridit (estimate & p-value)						-0.1544	-0.0815	-0.1199	-0.0298	-0.1108
						p = < .0001	p = 0.0015	p = < .0001	p = 0.1822	p = < .0001
Time*ridit (estimate & p-value)					0.0024					-0.0047
					p = 0.2133					p = 0.0176
Time*ridit*gender (estimate & p-value)										-0.0284
										p = < .0001
SII	0.215	0.257	0.259	0.291	0.295	0.179	0.238	0.282	0.322	0.342
95% CI	0.204–0.227	0.246–2.267	0.250–0.269	0.281–0.201	0.285–0.306	0.169–0.188	0.229–0.249	0.273–0.291	0.311–0.331	0.332–0.352
Gender*ridit (estimate & p-value)						0.0367	0.0184	-0.0225	-0.0301	-0.0467
						p = < .0001	p = 0.011	p = 0.0008	p = < .0001	p = < .0001
Time*ridit (estimate & p-value)					0.0064					0.0127
					p = < .0001					p = < .0001
Time*ridit*gender (estimate & p-value)										-0.0106
										p = < .0001

**Fig 3:**
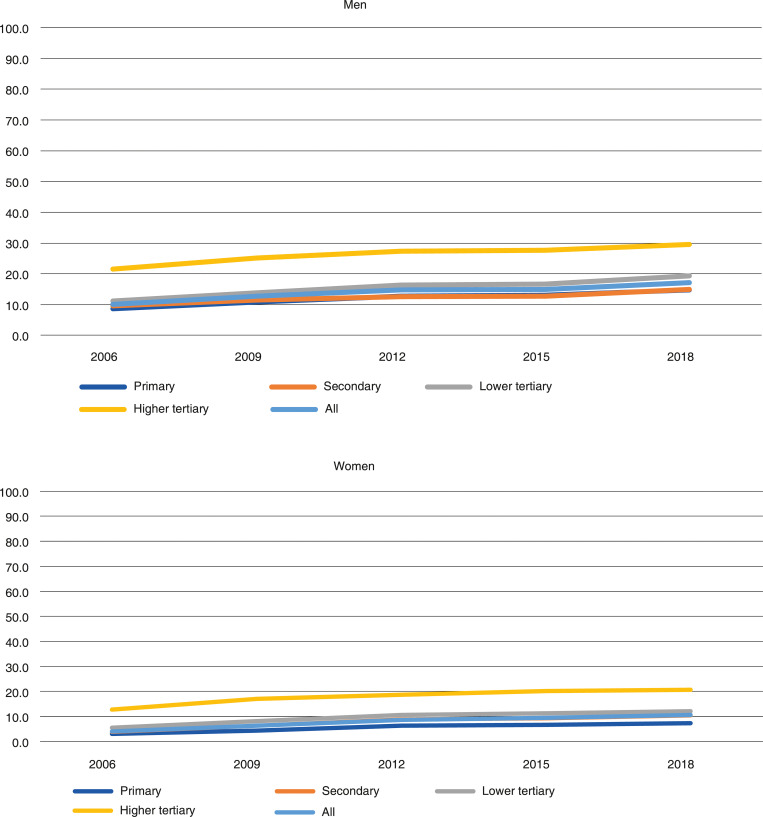
Employment rate of 66–68-year-old men and women in Finland in 2006–2018, by educational level.

Also, among the 66–68-year-olds, employment increased in all educational classes over the period 2006–2018 (Fig 3). In this age-group, the employment rates were relatively low. In 2006, it was 9 percent among men and 3 percent among women with a primary education. In 2018, the equivalent figures were 15 and 7 percent. Among women with a higher tertiary education, the employment rate was 13 percent in 2006 and 21 percent in 2018. The corresponding figures for men were 21 and 30 percent.

Among the 66–68-year-olds, educational inequalities were again evident (Table 4), although the SII were considerably lower than in the younger age groups. The latter is due to the overall low employment rates in the older age bracket. The low employment rates are also reflected in smaller differences in absolute inequalities between men and women: since 2012 there were no statistically significant gender differences in the SII. Gender differences in relative inequalities also narrowed somewhat but continued to exist, with RII values for women substantially higher than for men throughout the observation period. While men’s relative inequalities remained fairly stable between 2006 and 2018, the RII among women declined from 3.86 in 2009 to 2.80 in 2018. Like for the younger age groups, absolute educational inequalities in employment increased for the 66–68-year-olds between 2006 and 2018. Again, the increase in SII was larger among women during this period, from 0.046 to 0.101.

**Table 4:** Educational inequalities in employment among 66–68-year-old men and women. Relative index of inequality (RII) and Slope index of inequality (SII) and 95-percent confidence intervals (95% CI).

	Men					Women				
	2006	2009	2012	2015	2018	2006	2009	2012	2015	2018
RII	2.17	2.27	2.09	2.08	2.10	3.34	3.86	3.00	3.02	2.80
95% CI	2.00–2.36	2.12–2.44	1.97–2.21	1.98–2.20	1.99–2.21	2.94–3.80	3.50–4.26	2.79–3.22	2.83–3.22	2.62–2.99
Gender*ridit (estimate & p-value)						-0.04297	-0.5276	-0.3634	-0.3714	-0.2892
						p = < .0001	p = < .0001	p = < .0001	p = < .0001	p = < .0001
Time*ridit (estimate & p-value)					-0.0057					-0.0233
					p = 0.0975					p = < .0001
Time*ridit*gender (estimate & p-value)										-0.0388
										p = < .0001
SII	0.075	0.096	0.097	0.097	0.111	0.046	0.079	0.090	0.097	0.101
95% CI	0.066–0.084	0.087–0.105	0.089–0.105	0.089–0.104	0.103–0.120	0.041–0.051	0.073–0.085	0.084–0.097	0.091–0.103	0.095–0.108
Gender*ridit (p-value)						0.0291	0.0173	0.0068	-0.0005	0.0096
						p = < .0001	p = 0.0015	p = 0.1889	p = 0.9140	p = 0.0763
Time*ridit (p-value)					0.0023					0.0042
					p = < .0001					p = < .0001
Time*ridit*gender (estimate & p-value)										-0.0008
										p = 0.0038

Additional results (see S2 and S4 Tables in S1 File) showed that a lower earnings limit for employment (2,000 euros in 2006) increased the employment rate in all educational classes, while a higher earnings limit for employment (8,000 euros in 2006) lowered the employment rates somewhat. Changes in the definition of employment affected the employment of those with a primary or secondary education the most. This means that, in these groups, there are more people with low incomes. With a lower earnings limit, more people are assessed as employed and therefore the employment rate grows especially among those with a low educational class. In addition, a lower limit for employment led to smaller educational inequalities in employment while a higher limit for employment to larger educational inequalities in all age-groups and for both men and women. However, time trends in educational inequalities in employment were relatively similar in all euro limits for employment among those aged 60–62 years. Among men and women aged 63–65, the RII narrowed, increased or remained stable over the study period depending on how employment was defined (see Table 3, S3 and S5 Tables in S1 File). However, changes in trends in RII were very small. Trends in SII were similar with all euro limits for employment. In addition, among those aged 66–68, the trends in RII and SII were relatively similar with all earnings limits.

## Discussion

In this study we aimed to present an overall picture of the employment of 60–68-year-old people by education and to study educational inequalities in employment in three age groups aged 60–62, 63–65 and 66–68 in Finland over the study period 2006–2018. We studied the relative and absolute educational inequalities in employment separately. Previous knowledge about the employment of people near and beyond the old-age retirement age and educational inequalities in employment is scarce. With our exceptional total register data it was possible to get an overall picture of the employment of the population near and beyond the old-age retirement age by educational levels over the period 2006–2018. As far as we know, this is the first study in this field and a novel method to study educational inequalities in employment.

Lengthening working careers and postponing retirement have been major policy aims in many western countries in recent years. Improving the employment rate of people near or beyond the old-age retirement age has been topical in Finland as well as in other counties. At same time educational inequalities in employment cause worries. An essential question is whether employment has developed similarly on all educational levels. Our study found that the employment rate increased in all age groups (60–62, 63–65 and 66–68 years) and on all educational levels for men and women over the period 2006–2018. The employment rate was strongly associated with age. Employment rates were the highest among the 60–62-year-old men and women with a higher tertiary education (over 80% for both genders in 2018) and the lowest among the 66–68-year-old women with a primary education (15% for men and 7% for women in 2018).

Relative educational inequalities in employment, measured by the RII, were present in all age groups and for both genders. During the study period, the RII varied from under two to over three, indicating that the employment rate of the high-educated persons was two to three times that of the low-educated persons. Also, absolute educational inequalities in employment, measured by the SII, were found in all age groups and for both genders. However, the magnitude of these inequalities varied substantially, mostly depending on the level of employment. The SII varied from under 0.1 to almost 0.4. In other words, the employment rate of the high-educated was 10 to 40 percentage points higher than that of the low-educated.

During the period under study, relative educational inequalities in employment mainly narrowed, with few exceptions. In contrast, absolute educational inequalities in employment mainly increased during the inspection period. Nevertheless, throughout the study period, relative educational inequalities were lowest among 60–62-year-old men and women who had the highest employment rates overall. At the same time, absolute educational inequalities were highest in this age group. Relative educational inequalities, in turn, were highest among the 66–68-year-old women who had the lowest employment rates overall. At the same time, absolute educational inequalities were lowest in this age group.

Among the 60–62-year-olds, relative educational inequalities in employment narrowed for both men and women. There may be many reasons for narrowed relative educational inequalities. At first, early exit from the labour market via the disability pension and other exit pathways have decreased over the study period in Finland [29, 66, 67]. The decrease has been the highest among those with a primary or a secondary education and has concerned mainly full disability pensions [66]. On the other hand, the incidence of partial disability pension has grown especially among those with secondary and lower tertiary education [66]. This may also affect the employment of those aged 60–62 years since those who retire on partial disability benefits usually continue working and tend to be over 55 years old [59]. In addition, unemployment in the age-group 60–62 has declined. It is known that the incidence of unemployment is more common among those with a lower than those with a higher education [68]. Absolute educational inequalities in employment showed some increase, but that can partly be explained by the fact that the employment level increased over the study period.

In the age group 63–65 years, relative educational inequalities were considerably stable among men and rather stable among women. A previous Finnish study with follow-up data from 2008 to 2016 has shown that the highly educated remain more often in employment beyond age 63 while those with a lower socioeconomic status are less willing to continue working longer [54]. Some of the people in this age group have already retired on an old-age pension and may therefore also be working alongside the pension. Previous studies on working while drawing an old-age pension have shown educational inequalities in post-retirement working [8, 49, 51, 52]. Our study concurs with these results and shows relatively high and rather stable educational inequalities in employment between 2006–2018 among those aged 63–65. Obviously, health and working conditions play an important role in the retirement and employment decision, especially among the low-educated [24, 54]. Also in this age group, absolute educational inequalities increased somewhat, but that can partly be explained by the fact that employment has increased over the study period and therefore absolute educational inequalities have grown.

Among the 66–68-year-old men, relative educational inequalities in employment remained quite stable over the study period. Among women, they narrowed. In this age group, the majority have already retired on an old-age pension. Therefore, in most cases, employment in this age group means working alongside a pension. Our results are in line with previous studies of post-retirement employment [2, 4–6, 8, 48, 50, 69, 70]. Working in old-age retirement is more common among those with a higher tertiary education compared to other educational levels. The relatively high employment rate among those with a higher tertiary education compared to others in this age group may be due to better health and physically less demanding working conditions, as well as the nature of work of those with a higher education and, consequently, their improved opportunities to remain employed at older ages. Absolute educational inequalities widened in this age group over the study period. This can be explained by the increased employment rate.

Educational inequalities in employment were rather different for men than women. Generally, women seemed to have larger educational inequalities in employment than men. Larger educational inequalities in employment among older women may be partly due to the relatively low employment rate of low-educated women. Employment rates have increased quite fast especially among 60–62-year-old women with secondary and lower tertiary education, but also among 66–68-year-old low-educated women (See Figs 1–3). This may be one reason why relative educational inequalities have narrowed especially among 60–62- and 66–68-year-old women.

Both relative and absolute inequalities are important to study when examining educational inequalities in employment over time. Our study found that trends in educational inequalities in employment over the study period differed depending on whether the inequalities were measured in absolute or relative terms. The differences between absolute and relative educational inequalities in employment can be explained by the fact that the size of the absolute inequalities may vary even if the relative inequalities remain constant [61, 62, 65, 71]. Relative inequalities highlight inequality per se. Absolute educational inequalities are more dependent on the employment level than relative inequalities. If the employment rate is low, there may be higher relative inequalities even if the absolute inequalities are small. If the employment rate grows, the relative inequalities may narrow even if the absolute inequalities grow.

A major strength of our study is that our data come from reliable registers with no missing information. We used income as an indicator of employment. For those who are retired, information on their labour force participation is otherwise difficult to define. We used a 5,000-euro limit for employment (in 2006) in our main analysis. This limit can be seen to represent a relatively good annual income from work or self-employment. In addition, we used lower and higher earnings limits to estimate how sensitive our results were. However, a certain income from employment means a different kind of labour input for different people. This can be seen as a limitation because it does not necessarily reveal the amount of working or working hours. For example, a high-educated person may work as a consultant for two weeks during a calendar year, whereas a low-educated person may work for several months; yet their earnings are the same. This type of inequality is not revealed in our analysis but can be identified as a subject for further research.

To conclude, our study found that employment increased among the 60–68-year-old men and women on all educational levels over the study period. We found educational inequalities in the employment of older people but no strong evidence for increased educational inequalities. Educational inequalities in the employment of those aged over 60 will continue to be an essential topic in the upcoming years, as the statutory retirement age is rising in many countries. It is important to study this subject with versatile methods to gain a comprehensive understanding of the educational inequalities in the employment of older people. Moreover, educational expansion has contributed to increasing employment among older workers as a type of invisible policy instrument. However, as educational expansion will eventually reach its limits, so will its effect on employment in the future. In addition, more studies are needed on how other factors, for example working conditions or health, explain the educational inequalities in employment of older people. Policies to improve the employment of older workers will need to increasingly focus on further decreasing the gap between the low-educated and the high-educated.

## Supporting information

S1 File (DOCX)
