# Supplementary material for: Educational inequalities in employment of Finns aged 60–68 in 2006–2018
Source: PLoS One. 2022 Oct 17;17(10):e0276003. doi: 10.1371/journal.pone.0276003 (PMC9576099; doi:10.1371/journal.pone.0276003)
Supplement: S1 File — (DOCX) [file pone.0276003.s001.docx]

**Supporting information**

**S1 Table. Distribution of educational level in 2006-2018, by age and gender**

|  |  |  | 2006 | 2009 | 2012 | 2015 | 2018 |
| --- | --- | --- | --- | --- | --- | --- | --- |
|  |  |  |  |  |  |  |  |
| Men | Age | Primary | 0.41 | 0.37 | 0.31 | 0.25 | 0.22 |
|  | 60–62 | Secondary | 0.31 | 0.35 | 0.41 | 0.44 | 0.46 |
|  |  | Lower tertiary | 0.20 | 0.20 | 0.20 | 0.21 | 0.21 |
|  |  | Higher tertiary | 0.08 | 0.08 | 0.08 | 0.09 | 0.11 |
|  |  |  |  |  |  |  |  |
|  |  |  |  |  |  |  |  |
|  |  |  |  |  |  |  |  |
|  | Age | Primary | 0.46 | 0.41 | 0.37 | 0.31 | 0.24 |
|  | 63–65 | Secondary | 0.27 | 0.31 | 0.35 | 0.40 | 0.45 |
|  |  | Lower tertiary | 0.19 | 0.20 | 0.20 | 0.20 | 0.22 |
|  |  | Higher tertiary | 0.08 | 0.08 | 0.08 | 0.09 | 0.10 |
|  |  |  |  |  |  |  |  |
|  |  |  |  |  |  |  |  |
|  |  |  |  |  |  |  |  |
|  | Age | Primary | 0.54 | 0.46 | 0.41 | 0.36 | 0.30 |
|  | 66–68 | Secondary | 0.24 | 0.27 | 0.31 | 0.35 | 0.40 |
|  |  | Lower tertiary | 0.16 | 0.19 | 0.20 | 0.20 | 0.20 |
|  |  | Higher tertiary | 0.06 | 0.08 | 0.09 | 0.08 | 0.09 |
|  |  |  |  |  |  |  |  |
|  |  |  |  |  |  |  |  |
|  |  |  |  |  |  |  |  |
| Women | Age | Primary | 0.41 | 0.35 | 0.28 | 0.21 | 0.16 |
|  | 60–62 | Secondary | 0.33 | 0.37 | 0.42 | 0.43 | 0.43 |
|  |  | Lower tertiary | 0.20 | 0.22 | 0.24 | 0.27 | 0.30 |
|  |  | Higher tertiary | 0.06 | 0.06 | 0.07 | 0.09 | 0.11 |
|  |  |  |  |  |  |  |  |
|  |  |  |  |  |  |  |  |
|  |  |  |  |  |  |  |  |
|  | Age | Primary | 0.47 | 0.41 | 0.35 | 0.28 | 0.20 |
|  | 63–65 | Secondary | 0.30 | 0.33 | 0.37 | 0.41 | 0.43 |
|  |  | Lower tertiary | 0.18 | 0.20 | 0.22 | 0.24 | 0.27 |
|  |  | Higher tertiary | 0.05 | 0.06 | 0.06 | 0.07 | 0.09 |
|  |  |  |  |  |  |  |  |
|  |  |  |  |  |  |  |  |
|  |  |  |  |  |  |  |  |
|  | Age | Primary | 0.55 | 0.47 | 0.40 | 0.35 | 0.28 |
|  | 66–68 | Secondary | 0.27 | 0.30 | 0.33 | 0.37 | 0.41 |
|  |  | Lower tertiary | 0.14 | 0.18 | 0.20 | 0.22 | 0.24 |
|  |  | Higher tertiary | 0.03 | 0.05 | 0.06 | 0.06 | 0.07 |
|  |  |  |  |  |  |  |  |
|  |  |  |  |  |  |  |  |

**S2 Table. Employment rate (%) of 60–62-, 63–65 and 66–68-year-old men and women by educational level in Finland, 2006-2018. Limit for employment=2000 euros in 2006**

|  |  | 2006 | 2009 | 2012 | 2015 | 2018 |
| --- | --- | --- | --- | --- | --- | --- |
|  |  |  |  |  |  |  |
| Men | Primary | 47 | 48 | 50 | 51 | 56 |
| 60–62 | Secondary | 51 | 52 | 56 | 58 | 64 |
|  | Lower tertiary | 63 | 66 | 70 | 71 | 76 |
|  | Higher tertiary | 79 | 79 | 82 | 81 | 82 |
|  | All | 54 | 55 | 59 | 61 | 67 |
|  |  |  |  |  |  |  |
| Women | Primary | 43 | 44 | 49 | 51 | 53 |
| 60–62 | Secondary | 50 | 55 | 63 | 65 | 70 |
|  | Lower tertiary | 62 | 67 | 74 | 77 | 80 |
|  | Higher tertiary | 78 | 79 | 82 | 82 | 84 |
|  | All | 51 | 55 | 63 | 67 | 72 |
|  |  |  |  |  |  |  |
|  |  |  |  |  |  |  |
| Men | Primary | 27 | 32 | 31 | 32 | 34 |
| 63–65 | Secondary | 29 | 34 | 34 | 36 | 38 |
|  | Lower tertiary | 37 | 44 | 44 | 47 | 49 |
|  | Higher tertiary | 53 | 62 | 61 | 63 | 63 |
|  | All | 32 | 38 | 37 | 39 | 42 |
|  |  |  |  |  |  |  |
|  |  |  |  |  |  |  |
| Woman | Primary | 19 | 25 | 25 | 28 | 27 |
| 63–65 | Secondary | 23 | 31 | 35 | 39 | 39 |
|  | Lower tertiary | 27 | 36 | 41 | 46 | 48 |
|  | Higher tertiary | 48 | 57 | 58 | 61 | 60 |
|  | All | 23 | 31 | 34 | 39 | 41 |
|  |  |  |  |  |  |  |
|  |  |  |  |  |  |  |
| Men | Primary | 14 | 17 | 18 | 19 | 20 |
| 66–68 | Secondary | 15 | 17 | 18 | 18 | 20 |
|  | Lower tertiary | 17 | 20 | 22 | 23 | 25 |
|  | Higher tertiary | 28 | 33 | 34 | 34 | 35 |
|  | All | 15 | 19 | 20 | 21 | 22 |
|  |  |  |  |  |  |  |
| Woman | Primary | 6 | 8 | 11 | 11 | 11 |
| 66–68 | Secondary | 7 | 10 | 14 | 15 | 15 |
|  | Lower tertiary | 9 | 13 | 16 | 16 | 17 |
|  | Higher tertiary | 18 | 23 | 25 | 25 | 26 |
|  | All | 7 | 10 | 14 | 14 | 15 |
|  |  |  |  |  |  |  |

**S3 Table. Educational inequalities in employment among 60–62-, 63–65- and 66–68-year-old men and women. Relative index of inequality (RII) and Slope index of inequality (SII) and 95-percent confidence intervals (95% CI). Limit for employment=2000 euros in 2006**

|  |  | Men | | | | |  | Women | | | | |
| --- | --- | --- | --- | --- | --- | --- | --- | --- | --- | --- | --- | --- |
|  |  | 2006 | 2009 | 2012 | 2015 | 2018 |  | 2006 | 2009 | 2012 | 2015 | 2018 |
|  |  |  |  |  |  |  |  |  |  |  |  |  |
| 60-62 year | RII | 1.84 | 1.86 | 1.84 | 1.78 | 1.63 |  | 1.97 | 1.99 | 1.79 | 1.69 | 1.58 |
|  | 95% CI | 1.80-1.88 | 1.83-1.90 | 1.81-1.87 | 1.75-1.81 | 1.60-1.66 |  | 1.93-2.01 | 1.95-2.03 | 1.76-1.81 | 1.66-1.71 | 1.56-1.60 |
|  |  |  |  |  |  |  |  |  |  |  |  |  |
|  | Gender*ridit (estimate & p-value) |  |  |  |  |  |  | -0.0659 | -0.0639 | 0.0300 | 0.0522 | 0.0299 |
|  |  |  |  |  |  |  |  | p=<.0001 | p=<.0001 | p=0.0129 | p=<.0001 | p=0.0039 |
|  | Time*ridit (estimate & p-value) |  |  |  |  | -0.0106 |  |  |  |  |  | -0.0218 |
|  |  |  |  |  |  | p=<.0001 |  |  |  |  |  | p=<.0001 |
|  | Time*ridit*gender (estimate & p-value) |  |  |  |  |  |  |  |  |  |  | -0.0102 |
|  |  |  |  |  |  |  |  |  |  |  |  | p=<.0001 |
|  |  |  |  |  |  |  |  |  |  |  |  |  |
|  |  |  |  |  |  |  |  |  |  |  |  |  |
|  | SII | 0.314 | 0.330 | 0.347 | 0.344 | 0.326 |  | 0.336 | 0.375 | 0.370 | 0.361 | 0.341 |
|  | 95% CI | 0.303-0.325 | 0.319-0.340 | 0.337-0.358 | 0.334-0.354 | 0.315-0.336 |  | 0.325-0.346 | 0.365-0.385 | 0.361-0.380 | 0.351-0.370 | 0.332-0.351 |
|  |  |  |  |  |  |  |  |  |  |  |  |  |
|  | Gender*ridit (estimate & p-value) |  |  |  |  |  |  | -0.0214 | -0.0453 | -0.0226 | -0.0167 | -0.0155 |
|  |  |  |  |  |  |  |  | p=0.0067 | p=<.0001 | p=0.0019 | p=0.0200 | p=0.0293 |
|  | Time*ridit (estimate & p-value) |  |  |  |  | 0.0015 |  |  |  |  |  | -0.0012 |
|  |  |  |  |  |  | p=0.0091 |  |  |  |  |  | p=0.0238 |
|  | Time*ridit*gender (estimate & p-value) |  |  |  |  |  |  |  |  |  |  | -0.0088 |
|  |  |  |  |  |  |  |  |  |  |  |  | p=<.0001 |
|  |  |  |  |  |  |  |  |  |  |  |  |  |
|  |  |  |  |  |  |  |  |  |  |  |  |  |
| 63-65 year | RII | 2.04 | 2.05 | 2.14 | 2.21 | 2.12 |  | 2.37 | 2.23 | 2.38 | 2.31 | 2.40 |
|  | 95% CI | 1.96-2.12 | 1.99-2.11 | 2.08-2.20 | 2.15-2.27 | 2.06-2.18 |  | 2.27-2.49 | 2.17-2.13 | 2.31-2.45 | 2.24-2.37 | 2.33-2.46 |
|  |  |  |  |  |  |  |  |  |  |  |  |  |
|  | Gender*ridit (estimate & p-value) |  |  |  |  |  |  | -0.1525 | -0.0906 | -0.1053 | -0.044 | -0.1207 |
|  |  |  |  |  |  |  |  | p=<.0001 | p=<.0001 | p=<.0001 | p=0.0256 | p=<.0001 |
|  | Time*ridit (estimate & p-value) |  |  |  |  | 0.0045 |  |  |  |  |  | -0.0016 |
|  |  |  |  |  |  | p=0.0071 |  |  |  |  |  | p=0.3619 |
|  | Time*ridit*gender (estimate & p-value) |  |  |  |  |  |  |  |  |  |  | -0.0270 |
|  |  |  |  |  |  |  |  |  |  |  |  | p=<.0001 |
|  |  |  |  |  |  |  |  |  |  |  |  |  |
|  |  |  |  |  |  |  |  |  |  |  |  |  |
|  |  |  |  |  |  |  |  |  |  |  |  |  |
|  | SII | 0.214 | 0.250 | 0.259 | 0.285 | 0.290 |  | 0.189 | 0.242 | 0.288 | 0.321 | 0.348 |
|  | 95% CI | 0.202-0.226 | 0.240-0.261 | 0.249-0.269 | 0.275-0.296 | 0.279-0.302 |  | 0.179-0.200 | 0.232-0.252 | 0.278-0.298 | 0.311-0.331 | 0.337-0.358 |
|  |  |  |  |  |  |  |  |  |  |  |  |  |
|  | Gender*ridit (estimate & p-value) |  |  |  |  |  |  | 0.0247 | 0.0082 | -0.0291 | -0.0354 | -0.0578 |
|  |  |  |  |  |  |  |  | p=0.0025 | p=0.2785 | p=<.0001 | p=<.0001 | p=<.0001 |
|  | Time*ridit (estimate & p-value) |  |  |  |  | 0.0060 |  |  |  |  |  | 0.0119 |
|  |  |  |  |  |  | p=<.0001 |  |  |  |  |  | p=<.0001 |
|  | Time*ridit*gender (estimate & p-value) |  |  |  |  |  |  |  |  |  |  | -0.0117 |
|  |  |  |  |  |  |  |  |  |  |  |  | p=<.0001 |
|  |  |  |  |  |  |  |  |  |  |  |  |  |
|  |  |  |  |  |  |  |  |  |  |  |  |  |
|  |  |  |  |  |  |  |  |  |  |  |  |  |
| 66-68 year | RII | 1.77 | 1.87 | 1.75 | 1.74 | 1.83 |  | 2.35 | 2.58 | 2.26 | 2.22 | 2.23 |
|  | 95% CI | 1.65-1.89 | 1.77-1.98 | 1.67-1.83 | 1.66-1.81 | 1.75-1.91 |  | 2.14-2.58 | 2.40-2.78 | 2.14-2.40 | 2.10-2.33 | 2.11-2.35 |
|  |  |  |  |  |  |  |  |  |  |  |  |  |
|  | Gender*ridit (estimate & p-value) |  |  |  |  |  |  | -0.2858 | -0.3198 | -0.2592 | -0.2433 | -0.199 |
|  |  |  |  |  |  |  |  | p=<.0001 | p=<.0001 | p=<.0001 | p=<.0001 | p=<.0001 |
|  | Time*ridit (estimate & p-value) |  |  |  |  | -0.0009 |  |  |  |  |  | -0.0123 |
|  |  |  |  |  |  | p=0.7361 |  |  |  |  |  | p=0.0005 |
|  | Time*ridit*gender (estimate & p-value) |  |  |  |  |  |  |  |  |  |  | -0.0350 |
|  |  |  |  |  |  |  |  |  |  |  |  | p=<.0001 |
|  |  |  |  |  |  |  |  |  |  |  |  |  |
|  |  |  |  |  |  |  |  |  |  |  |  |  |
|  |  |  |  |  |  |  |  |  |  |  |  |  |
|  | SII | 0.084 | 0.109 | 0.104 | 0.103 | 0.121 |  | 0.060 | 0.094 | 0.107 | 0.110 | 0.118 |
|  | 95% CI | 0.074-0.095 | 0.099-0.120 | 0.095-0.114 | 0.094-0.112 | 0.111-0.130 |  | 0.054-0.068 | 0.086-0.102 | 0.099-0.114 | 0.103-0.117 | 0.110-0.125 |
|  |  |  |  |  |  |  |  |  |  |  |  |  |
|  | Gender*ridit (estimate & p-value) |  |  |  |  |  |  | 0.0234 | 0.0161 | -0.0022 | -0.0072 | 0.0029 |
|  |  |  |  |  |  |  |  | p=0.0003 | p=0.0142 | p=0.7157 | p=0.2098 | p=0.6409 |
|  | Time*ridit (estimate & p-value) |  |  |  |  | 0.0020 |  |  |  |  |  | 0.0040 |
|  |  |  |  |  |  | p=0.0002 |  |  |  |  |  | p=<.0001 |
|  | Time*ridit*gender (estimate & p-value) |  |  |  |  |  |  |  |  |  |  | -0.0030 |
|  |  |  |  |  |  |  |  |  |  |  |  | p=<.0001 |

**S4 Table. Employment rate of 60–62-, 63–65- and 66–68-year-old men and women by educational level in Finland, 2006-2018. Limit for employment=8000 euros in 2006.**

|  |  | 2006 | 2009 | 2012 | 2015 | 2018 |
| --- | --- | --- | --- | --- | --- | --- |
|  |  |  |  |  |  |  |
| Men | Primary | 39 | 39 | 42 | 43 | 47 |
| 60-62 | Secondary | 43 | 44 | 48 | 49 | 55 |
|  | Lower tertiary | 57 | 59 | 64 | 65 | 69 |
|  | Higher tertiary | 75 | 75 | 78 | 77 | 78 |
|  | All | 47 | 48 | 52 | 53 | 58 |
|  |  |  |  |  |  |  |
| Women | Primary | 36 | 37 | 42 | 43 | 43 |
| 60-62 | Secondary | 42 | 47 | 55 | 57 | 61 |
|  | Lower tertiary | 55 | 61 | 68 | 71 | 73 |
|  | Higher tertiary | 74 | 76 | 78 | 78 | 80 |
|  | All | 44 | 48 | 56 | 60 | 64 |
|  |  |  |  |  |  |  |
| Men | Primary | 18 | 22 | 21 | 22 | 24 |
| 63-65 | Secondary | 21 | 25 | 24 | 25 | 28 |
|  | Lower tertiary | 28 | 35 | 35 | 38 | 40 |
|  | Higher tertiary | 45 | 54 | 54 | 56 | 55 |
|  | All | 23 | 28 | 28 | 29 | 32 |
|  |  |  |  |  |  |  |
| Woman | Primary | 12 | 17 | 17 | 18 | 18 |
| 63-65 | Secondary | 15 | 22 | 25 | 27 | 28 |
|  | Lower tertiary | 19 | 28 | 32 | 36 | 38 |
|  | Higher tertiary | 41 | 50 | 51 | 54 | 53 |
|  | All | 16 | 23 | 25 | 29 | 31 |
|  |  |  |  |  |  |  |
|  |  |  |  |  |  |  |
| Men | Primary | 6 | 8 | 10 | 9 | 11 |
| 66-68 | Secondary | 7 | 9 | 10 | 9 | 11 |
|  | Lower tertiary | 8 | 11 | 13 | 13 | 14 |
|  | Higher tertiary | 17 | 22 | 24 | 24 | 25 |
|  | All | 8 | 10 | 12 | 11 | 13 |
|  |  |  |  |  |  |  |
| Woman | Primary | 2 | 3 | 4 | 4 | 5 |
| 66-68 | Secondary | 3 | 4 | 6 | 6 | 7 |
|  | Lower tertiary | 4 | 6 | 8 | 8 | 8 |
|  | Higher tertiary | 10 | 14 | 16 | 17 | 17 |
|  | All | 3 | 4 | 6 | 7 | 7 |

**S5 Table. Educational inequalities in employment among 60–­62-, 63–65- and 66–68-year-old men and women. Relative index of inequality (RII) and Slope index of inequality (SII) and 95-percent confidence intervals (95% CI). Limit for employment, 8000 euros in 2006**

|  |  | Men | | | | |  | Women | | | | |
| --- | --- | --- | --- | --- | --- | --- | --- | --- | --- | --- | --- | --- |
|  |  | 2006 | 2009 | 2012 | 2015 | 2018 |  | 2006 | 2009 | 2012 | 2015 | 2018 |
|  |  |  |  |  |  |  |  |  |  |  |  |  |
| **60-62 year** | RII | 2.20 | 2.25 | 2.18 | 2.11 | 1.91 |  | 2.27 | 2.30 | 2.03 | 1.95 | 1.87 |
|  | 95% CI | 2.14-2.25 | 2.20-2.30 | 2.14-2.23 | 2.07-2.15 | 1.87-1.94 |  | 2.21-2.33 | 2.25-2.34 | 2.00-2.07 | 1.92-1.98 | 1.84-1.90 |
|  |  |  |  |  |  |  |  |  |  |  |  |  |
|  | Gender*ridit (estimate & p-value) |  |  |  |  |  |  | -0.0329 | -0.0212 | 0.0712 | 0.077 | 0.0194 |
|  |  |  |  |  |  |  |  | p=0.0634 | p=0.1712 | p=<.0001 | p=<.0001 | p=0.1230 |
|  | Time*ridit (estimate & p-value) |  |  |  |  | -0.0126 |  |  |  |  |  | -0.0202 |
|  |  |  |  |  |  | p=<.0001 |  |  |  |  |  | p=<.0001 |
|  | Time*ridit*gender (estimate & p-value) |  |  |  |  |  |  |  |  |  |  | -0.0125 |
|  |  |  |  |  |  |  |  |  |  |  |  | p=<.0001 |
|  |  |  |  |  |  |  |  |  |  |  |  |  |
|  |  |  |  |  |  |  |  |  |  |  |  |  |
|  | SII | 0.344 | 0.362 | 0.384 | 0.381 | 0.369 |  | 0.344 | 0.390 | 0.402 | 0.411 | 0.412 |
|  | 95% CI | 0.333-0.355 | 0.352-0.372 | 0.374-0.395 | 0.370-0.392 | 0.358-0.379 |  | 0.333-0.355 | 0.380-0.4000 | 0.392-0.412 | 0.401-0.421 | 0.402-0.422 |
|  |  |  |  |  |  |  |  |  |  |  |  |  |
|  | Gender*ridit (estimate & p-value) |  |  |  |  |  |  | 0.0001 | -0.0282 | -0.0182 | -0.0301 | -0.0435 |
|  |  |  |  |  |  |  |  | p=0.9931 | p=<.0001 | p=0.014 | p=<.0001 | p=<.0001 |
|  | Time*ridit (estimate & p-value) |  |  |  |  | 0.0025 |  |  |  |  |  | 0.0040 |
|  |  |  |  |  |  | p=<.0001 |  |  |  |  |  | p=<.0001 |
|  | Time*ridit*gender (estimate & p-value) |  |  |  |  |  |  |  |  |  |  | -0.0100 |
|  |  |  |  |  |  |  |  |  |  |  |  | p=<.0001 |
|  |  |  |  |  |  |  |  |  |  |  |  |  |
|  |  |  |  |  |  |  |  |  |  |  |  |  |
| **63-65 year** | RII | 2.72 | 2.79 | 2.90 | 3.11 | 2.89 |  | 3.19 | 2.93 | 3.27 | 3.22 | 3.29 |
|  | 95% CI | 2.59-2.85 | 2.69-2.89 | 2.80-3.00 | 3.00-3.22 | 2.79-2.99 |  | 3.02-3.39 | 2.81-3.05 | 3.15-3.39 | 3.11-3.33 | 3.18-3.41 |
|  |  |  |  |  |  |  |  |  |  |  |  |  |
|  | Gender*ridit (estimate & p-value) |  |  |  |  |  |  | -0.1626 | -0.0485 | -0.1205 | -0.0366 | -0.1312 |
|  |  |  |  |  |  |  |  | p=<.0001 | p=<.0001 | p=<.0001 | p=0.1437 | p=<.0001 |
|  | Time*ridit (estimate & p-value) |  |  |  |  | 0.0065 |  |  |  |  |  | 0.0015 |
|  |  |  |  |  |  | p=0.0021 |  |  |  |  |  | p=0.4874 |
|  | Time*ridit*gender (estimate & p-value) |  |  |  |  |  |  |  |  |  |  | -0.0294 |
|  |  |  |  |  |  |  |  |  |  |  |  | p=<.0001 |
|  |  |  |  |  |  |  |  |  |  |  |  |  |
|  |  |  |  |  |  |  |  |  |  |  |  |  |
|  | SII | 0.211 | 0.261 | 0.261 | 0.291 | 0.297 |  | 0.168 | 0.229 | 0.278 | 0.318 | 0.337 |
|  | 95% CI | 0.201-0.222 | 0.251-0.271 | 0.252-0.270 | 0.282-0.300 | 0.287-0.307 |  | 0.159-0.177 | 0.219-0.238 | 0.269-0.286 | 0.309-0.327 | 0.327-0.346 |
|  |  |  |  |  |  |  |  |  |  |  |  |  |
|  |  |  |  |  |  |  |  |  |  |  |  |  |
|  | Gender*ridit (estimate & p-value) |  |  |  |  |  |  | 0.0434 | 0.0326 | -0.0166 | -0.0269 | -0.0397 |
|  |  |  |  |  |  |  |  | p=<.0001 | p=<.0001 | p=0.0095 | p=<.0001 | p=<.0001 |
|  | Time*ridit (estimate & p-value) |  |  |  |  | 0.0065 |  |  |  |  |  | 0.0134 |
|  |  |  |  |  |  | p=<.0001 |  |  |  |  |  | p=<.0001 |
|  | Time*ridit*gender (estimate & p-value) |  |  |  |  |  |  |  |  |  |  | -0.0098 |
|  |  |  |  |  |  |  |  |  |  |  |  | p=<.0001 |
|  |  |  |  |  |  |  |  |  |  |  |  |  |
|  |  |  |  |  |  |  |  |  |  |  |  |  |
|  |  |  |  |  |  |  |  |  |  |  |  |  |
| **66-68 year** | RII | 2.37 | 2.69 | 2.41 | 2.62 | 2.54 |  | 4.72 | 4.98 | 3.91 | 4.25 | 3.87 |
|  | 95% CI | 2.15-2.61 | 2.48-2.92 | 2.26-2.57 | 2.46-2.79 | 2.39-2.71 |  | 4.01-5.57 | 4.41-5.62 | 0.58-4.27 | 3.92-4.61 | 3.56-4.22 |
|  |  |  |  |  |  |  |  |  |  |  |  |  |
|  | Gender*ridit (estimate & p-value) |  |  |  |  |  |  | -0.6873 | -0.6163 | -0.4836 | -0.4816 | -0.4210 |
|  |  |  |  |  |  |  |  | p=<.0001 | p=<.0001 | p=<.0001 | p=<.0001 | p=<.0001 |
|  | Time*ridit (estimate & p-value) |  |  |  |  | 0.002 |  |  |  |  |  | -0.0200 |
|  |  |  |  |  |  | p=0.6183 |  |  |  |  |  | p=0.0004 |
|  | Time*ridit*gender (estimate & p-value) |  |  |  |  |  |  |  |  |  |  | -0.0406 |
|  |  |  |  |  |  |  |  |  |  |  |  | p=<.0001 |
|  |  |  |  |  |  |  |  |  |  |  |  |  |
|  |  |  |  |  |  |  |  |  |  |  |  |  |
|  | SII | 0.062 | 0.087 | 0.088 | 0.091 | 0.099 |  | 0.036 | 0.064 | 0.078 | 0.084 | 0.084 |
|  | 95% CI | 0.054-0.069 | 0.079-0.095 | 0.081-0.096 | 0.085-0.098 | 0.092-0.106 |  | 0.032-0.041 | 0.059-0.069 | 0.073-0.083 | 0.080-0.089 | 0.079-0.089 |
|  |  |  |  |  |  |  |  |  |  |  |  |  |
|  | Gender*ridit (estimate & p-value) |  |  |  |  |  |  | 0.0254 | 0.0226 | 0.0104 | 0.0069 | 0.0150 |
|  |  |  |  |  |  |  |  | p=<.0001 | p=<.0001 | p=0.0212 | p=0.0974 | p=0.001 |
|  | Time*ridit (estimate & p-value) |  |  |  |  | 0.0025 |  |  |  |  |  | 0.0038 |
|  |  |  |  |  |  | p=<.0001 |  |  |  |  |  | p=<.0001 |
|  | Time*ridit*gender (estimate & p-value) |  |  |  |  |  |  |  |  |  |  | -0.0007 |
|  |  |  |  |  |  |  |  |  |  |  |  | p=<.0001 |
|  |  |  |  |  |  |  |  |  |  |  |  |  |
|  |  |  |  |  |  |  |  |  |  |  |  |  |
